# Effects of Oxide Additives on the Phase Structures and Electrical Properties of SrBi_4_Ti_4_O_15_ High-Temperature Piezoelectric Ceramics

**DOI:** 10.3390/ma14206227

**Published:** 2021-10-19

**Authors:** Shaozhao Wang, Huajiang Zhou, Daowen Wu, Lang Li, Yu Chen

**Affiliations:** 1 School of Mechanical Engineering, Chengdu University, Chengdu 610106, China; wangsz0908@163.com (S.W.); zhouhj1996@163.com (H.Z.); wdw132812976202021@163.com (D.W.); 2 Key Laboratory of Deep Earth Science and Engineering, Ministry of Education, Sichuan University, Chengdu 610065, China; lilang@scu.edu.cn

**Keywords:** SrBi_4_Ti_4_O_15_, high-temperature piezoceramics, oxide additives, curie temperature, piezoelectric coefficient, ion substitution

## Abstract

In this work, SrBi_4_Ti_4_O_15_ (SBT) high-temperature piezoelectric ceramics with the addition of different oxides (Gd_2_O_3_, CeO_2_, MnO_2_ and Cr_2_O_3_) were fabricated by a conventional solid-state reaction route. The effects of oxide additives on the phase structures and electrical properties of the SBT ceramics were investigated. Firstly, X-ray diffraction analysis revealed that all these oxides-modified SBT ceramics prepared presented a single SrBi_4_Ti_4_O_15_ phase with orthorhombic symmetry and space group of *Bb*21*m*, the change in cell parameters indicated that these oxide additives had diffused into the crystalline lattice of SBT and formed solid solutions with it. The SBT ceramics with the addition of MnO_2_ achieved a high relative density of up to 97%. The temperature dependence of dielectric constant showed that the addition of Gd_2_O_3_ could increase the *T*_C_ of SBT. At a low frequency of 100 Hz, those dielectric loss peaks appearing around 500 °C were attributed to the space-charge relaxation as an extrinsic dielectric response. The synergetic doping of CeO_2_ and Cr_2_O_3_ could reduce the space-charge-induced dielectric relaxation of SBT. The piezoelectricity measurement and electro-mechanical resonance analysis found that Cr_2_O_3_ can significantly enhance both *d*_33_ and *k*_p_ of SBT, and produce a higher phase-angle maximum at resonance. Such an enhanced piezoelectricity was attributed to the further increased orthorhombic distortion after Ti^4+^ at B-site was substituted by Cr^3+^. Among these compositions, Sr_0.92_Gd_0.053_Bi_4_Ti_4_O_15_ + 0.2 wt% Cr_2_O_3_ (SGBT-Cr) presented the best electrical properties including *T*_C_ = 555 °C, *tan δ =* 0.4%, *k*_p_ = 6.35% and *d*_33_ = 28 pC/N, as well as a good thermally-stable piezoelectricity that the value of *d*_33_ was decreased by only 3.6% after being annealed at 500 °C for 4 h. Such advantages provided this material with potential applications in the high-stability piezoelectric sensors operated below 500 °C.

## 1. Introduction

Piezoelectric ceramics, which are a kind of synthetic polycrystalline ferroelectric material, have been used as sensing materials for many electrical devices such as ultrasonic transducers, vibration sensors and multi-layer actuators [1,2,3,4,5]. In recent years, the concern for the environmental pollution and people’s health highlights the main differences between the commercial lead-based piezoelectric ceramics (lead zirconate titanate-PZT) and the developed lead-free piezoelectric ceramics (such as calcium barium titanate-BTO, potassium sodium niobate-KNN, etc.) [6,7,8]. Bismuth-layered structure ferroelectrics (BLSFs, also called Aurivillius phase), with large spontaneous polarization and fatigue-free properties, are promising candidates for ferroelectric random access memories (FRAMs) [9]. The chemical formula of BLSFs can be described as (Bi_2_O_2_)^2+^ (A*_m_*_−1_B_m_O_3*m*+1_)^2−^, where *m* delegates to the number of octahedral layers in the perovskite layer between the bismuth oxide layers and values normally from 1 to 5. Such as Bi_2_WO_6_ (*m* = 1, *T*_C_ = 950 °C) [10], Bi_3_TiNbO_9_ (*m* = 2, *T*_C_ = 904 °C) [11], Bi_4_Ti_3_O_12_ (*m* = 3, *T*_C_ = 675 °C) [12], SrBi_4_Ti_4_O_15_ (*m* = 4, *T*_C_ = 520 °C) [13], Sr_2_Bi_4_Ti_5_O_18_ (*m* = 5, *T*_C_ = 267 °C) [14]. Because of high Curie temperature, BLSFs have received more and more attention due to the urgent need of high-temperature sensor, actuator, and transducer applications in recent years [15].

Among the Aurivillius family, SrBi_4_Ti_4_O_15_ (SBT) captures an orthorhombic symmetry with space group *A*21*am* at room temperature, including four perovskite-like TiO_6_ octahedron units stacked in between (Bi_2_O_2_)^2+^ layers [16]. However, some disadvantages associated with SBT such as difficulty in polarization, high leakage current, volatilization of the bismuth during sintering, and a low density and undesirable properties caused by the random arrangement of plate-like crystal grains [17], such as a tenuous piezoelectric activity (*d*_33_~10 pC/N) [18]. Over the last few decades, a large number of investigations focused on the modification of the electrical properties of SBT piezoceramics through the ionic substitution at A-site [19,20,21] or B-site [22,23,24]. Cao et al. [25] found a large enhancement of piezoelectric properties (*d*_33_ = 30 pC/N) in Mn-modified (B-site) SrBi_4_Ti_4_O_15_ as well as good thermal stability at elevated temperatures, while its *T*_C_ remains almost unchanged at ~530 °C. However, most reports on enhancing the ferroelectric and piezoelectric properties of SBT ceramics concentrate on A-site rather than B-site [26]. For example, the Curie temperature increased by doping SBT with Na^+^ and Pr^3+^ at A-site [27]. The dielectric constant and loss decreased, whereas the Curie temperature increased when Na^+^ and Nd^3+^ were substituted to A-site in SBT [17]. A-site cerium-modified SrBi_4_Ti_4_O_15_ ceramics showed a high stability of dielectric properties [28]. On the other hand, some oxide compounds like Cr_2_O_3_, MnO_2_ and U_3_O_8_ as additives have been also proved to play a notable effect on the physical and electrical properties of BLSF ceramics [29].

Among these modified SBT ceramics, the compositions with both high Curie temperature and high piezoelectric property at the same time were rarely reported, and most of the as-reported works focused on the modification of the SBT ceramics with one kind of element/oxide. In this work, two kinds of oxides (Gd_2_O_3_, CeO_2_, MnO_2_ and Cr_2_O_3_) were co-doped into the SBT ceramics, and after these the oxide-modified SBT piezoceramics were fabricated via a traditional solid-state reaction process; the effect of these oxides on the phase structure and electrical properties of SBT ceramics have been investigated in detail.

## 2. Materials and Methods

### 2.1. Sample Preparation

Highly purified metal oxides of SrCO_3_ (99%), Bi_2_O_3_ (99%), TiO_2_ (99%), CeO_2_ (99.99%) and Gd_2_O_3_ (99.99%) were weighed according to the stoichiometric formula of three designed compositions: SrBi_4_Ti_4_O_15_ (SBT), Sr_0.92_Gd_0.053_Bi_4_Ti_4_O_15_ (SGBT) and Sr_0.96_Ce_0.04_Bi_4_Ti_4_O_15_ (SCBT), respectively. These powders as starting materials were milled for 10 h under the condition of ethanol as a dissolvent and zirconium ball as a milling medium. The dried mixtures were calcined at 850 °C for 4 h, and then the calcined powders of SGBT and SCBT were divided into four equal parts according to their quality, respectively. Secondly, 0.2 wt% of CeO_2_ (99.99%), MnO_2_ (99%) and Cr_2_O_3_ (99%) were severally added into three parts of SGBT powders, while 0.2 wt% of Gd_2_O_3_ (99.99%), MnO_2_ (99%) and Cr_2_O_3_ (99%) were severally added into three parts of SCBT powders. Then, the mixtures of SBT, SGBT, SGBT-Ce, SGBT-Mn, SGBT-Cr, SCBT, SCBT-Gd, SCBT-Mn, SCBT-Cr were milled again in the same requirement. After drying, polyvinyl alcohol (PVA) as binder was added to the uniform mixture to form granules. The granules were pressed into pellets of 10 mm in diameter and 1 mm in thickness. After burning out PVA at 600 °C for 2 h, these pellets were sintered between 1100 °C and 1200 °C for 2 h in a sealed alumina crucible to obtain the ceramics with the maximum density.

### 2.2. Sample Characterization

The crystallographic structure of all sintered samples was determined by an X-ray diffractometer (DX2700, Dandong, China) employing Cu-Kα radiation (*λ* = 1.5418 Å). Meanwhile, the relative density of all sintered samples was calculated as the ratio of the apparent density measured by the Archimedes method to the theoretical density obtained from crystallographic structures (XRD). In order to measure the electrical properties, the samples were polished and fired with silver paste as the electrodes at 700 °C for 10 min. The dielectric constant (*ε*_r_) and loss tangent (*tan δ*) as a function of temperature were recorded using an LCR analyzer (TH2829A, Tonghui, China) attached to a programmable furnace. Samples were poled under a DC field of 6–10 kV/mm for 15 min in a silicone oil bath at 150 °C. The electrical impedance (|*Z*|) and phase angle (*θ*) as a function of frequency was measured using an impedance analyzer (TH2829A, Tonghui, China). The planar electromechanical coupling factor (*k*_p_), mechanical quality factor (*Q*_m_) and planar frequency constant (*N*_p_) were calculated by the IEEE standard. Thermal depoling behavior was investigated by annealing the polarized samples at different temperatures for 4 h, and then the piezoelectric charge coefficient (*d*_33_) was remeasured using a quasi-static *d*_33_ m (ZJ-6AN, IACAS, Beijing, China) when the samples were cooled to room temperature.

## 3. Results and Discussion

### 3.1. Phase Structure of Ceramics

**Figure 1:**
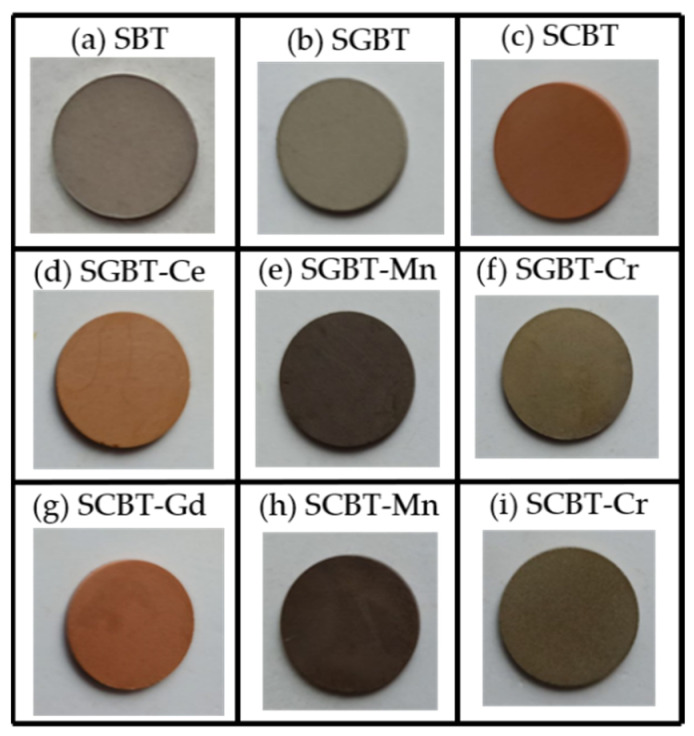
Appearances of the oxide-modified SBT ceramics (the corresponding chemical compositions (marked with (**a**–**i**) respectively) are located above the samples).

The appearances of the oxide-modified SBT piezoceramics are presented in Figure 1. As can be seen from these figures, the pure SBT ceramic seems to be taupe; after it was doped with different oxides, different colors were presented. However, all these oxide-modified SBT piezoceramics were sintered in a uniform color and free of cracks, blotches, striations and holes, at least seen from their surfaces. The change of color also proves that the oxides as additives have dissolved into SBT, leading different color emerging mechanisms to the ceramics.

**Figure 2:**
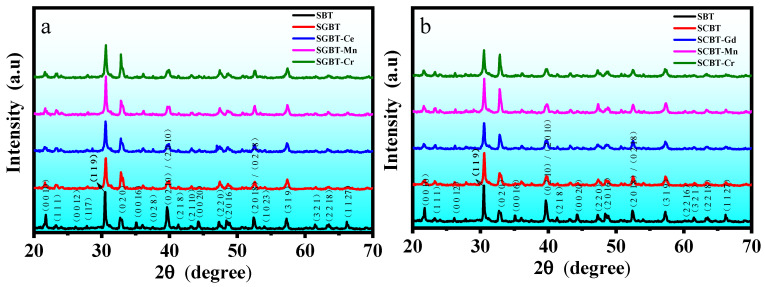
XRD patterns of the oxide-modified SBT ceramics: (**a**) SGBT-series; (**b**) SCBT-series.

The XRD patterns of the oxide-modified SBT piezoceramics are shown in Figure 2. It can be seen that these samples display a single SrBi_4_Ti_4_O_15_ phase crystallized in the orthorhombic structure with *Bb*21*m* (36) space group (JCPDS No: 43-0973). There is no impurity detected from XRD patterns, which indicates that these oxide additives have been incorporated into the crystal lattice of SrBi_4_Ti_4_O_15_. The strongest diffraction of all these samples appears at the (1 1 9) peak, stating the fact that SrBi_4_Ti_4_O_15_ belongs to the BLSF with the structure of four layer (*m* = 4) [30]. Some variations observed from the details of XRD patterns can be related with the lattice distortions of SBT caused by doping. By contrasting with the pure SBT, three diffraction peaks of the doped SBT: (0 0 10), (0 0 16) and (0 0 20) are weakened, which indicates that their grains orientating along the *c*-axis becomes fewer [31]. Inversely, the diffraction peaks of (2 0 0)/(0 2 0) are enhanced, which states that the number of grains oriented along the *a*-*b* plane increased.

The lattice parameters of the oxide-modified SBT ceramics are given in Table 1. The lattice parameters (*a*, *c* and *v*) of the oxide-modified SBT decrease, whereas the values of orthorhombic distortion (*b*/*a*) increase, which may be attributed to the ion-substitution effect caused by the addition of different oxides. The bismuth oxide layer is very strong and bismuth ion in the bismuth oxide layer are difficult to be substituted by other ions [32], meanwhile ions with similar ionic radius and same coordination number are more likely to be mutually substituted [31], consequently Sr^2+^ located at the A-site in the perovskite layers are substituted by Gd^3+^/Ce^4+^ with smaller ion radius. Ti^4+^ located at the B-site in the perovskite layers would be substituted by Mn^3+^ and Cr^3+^. The lattice distortion caused by ion substitution can result in the change of electrical properties for ferroelectric compounds [33]; the larger *b*/*a* value is, more distorted the lattice is. Among these compositions, the unit cell of SGBT-Cr has the largest orthorhombic distortion with a *b*/*a* value of 1.0024.

Table 2 lists the density of the oxide-modified SBT ceramics. The relative density of SBT is measured as 94.8%, which has been changed after the addition of different oxides. According to the results given by Table 2, the addition of CeO_2_ and MnO_2_ played a positive effect on the densifying process of the SBT ceramic during sintering.

### 3.2. Dielectric Properties of Ceramics

**Figure 3:**
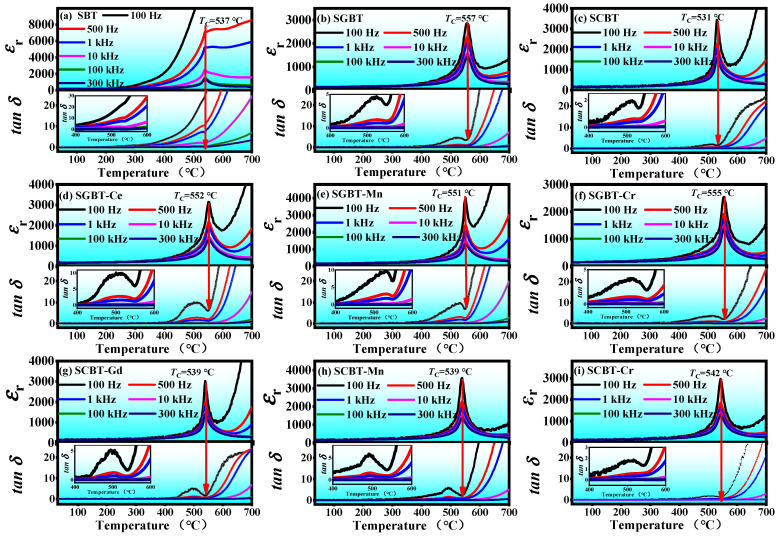
Temperature dependence of dielectric constant and loss tangent of the oxide-modified SBT ceramics at different frequencies.

Figure 3 exhibits the temperature dependence of dielectric constant (*ε*_r_) and loss tangent (*tan σ*) of the oxide-modified SBT ceramics. As can be seen, all the samples show a dielectric anomaly around 540 °C, which can be related to the ferroelectric-paraelectric phase transition of the ceramics. The peak position is considered as the Curie temperature (*T*_C_). For the pure SBT (*T*_C_ = 537 °C, Figure 3a), a sharp rise in the values of *ε*_r_ occurred above 350 °C at low frequencies (100 Hz and 500 Hz), which can be attributed to the dielectric response of a large number of space charges to the external electric field. Moreover, its permittivity peaks are broadened and strongly dependent with frequency in terms of strength, and their positions seem to be also dependent with frequency as marked by the slightly oblique arrows. Therefore, this can be considered as a typical relaxed dielectric behavior, which is partially due to the compositional fluctuation in the crystallographic sites. In Figure 3b, SGBT exhibits a higher *T*_C_ ~ 557 °C as well as a normal phase transition. This result may be attributed to the lattice distortion of the pseduo-perovskite structure since the bivalent strontium ions were substituted by the trivalent gadolinium ions at the *A*-site. The tolerance factor (*t*) which is used for evaluating the stability of ABO_3_-type perovskite structure can be calculated by the expression as follows [34]:

(1) $$t=\frac{r_{A}{+r}_{O}}{\sqrt{2}{(r}_{B}{+r}_{O})}$$

where *r*_A_, *r*_B_ and *r*_O_ are the ionic radius of A, B and the oxygen ion, respectively. One-third of bivalent strontium ions (1.44 Å) and two-thirds of bismuth ions (1.30 Å) occupy the A site at the perovskite-like structure of pure SBT ceramics. According to the atomic percentage of the A-/B-site, the average ionic radius for Sr_0.92_Gd_0.053_Bi_4_Ti_4_O_15_ could be reckoned as follows: *r*_A_ = 1/3 (0.92*r*_Sr2+_ + 0.053*r*_Gd3+_) + 2/3 *r*_Bi3+_ = 1.33 Å (*r*_Gd3+_ = 1.107 Å), *r*_B_ = *r*_Ti4+_ = 0.605 Å, *r*_O2−_ = 1.40 Å. The tolerance factor of SGBT and SBT were calculated to be 0.96 and 0.97, respectively, according to Equation (1). The reduced tolerance factor indicates that the perovskite structure of SGBT is more stable; in this case, the phase transition from the ferroelectric state to the paraelectric state needs more energy, which corresponds to a higher *T*_C_. As can be seen from Figure 3c, *T*_C_ of SCBT (531 °C) is less low than that of SBT, which could be attributed to the reduced stability of oxygen octahedron after adding CeO_2_ into SBT, since the coordination number of introduced Ce^4+^ is smaller than that of Sr^2+^. The dielectric loss peak appearing around 500 °C at the low frequency of 100 Hz could be attributed to the space-charge relaxation as an extrinsic dielectric response [35]. The similar dielectric anomaly was also observed in cobalt-modified SBT [23]. The defect dipoles which are formed by combining space charges or ions with opposite charges may be slow to follow the external electric field, thereby contributing to the dielectric loss [36]. Therefore, the relaxation phenomenon reflected by the dielectric loss peaks or bumps in the wide temperature sweep can be related to the viscoelastic reorientation of defect dipoles following the external electric field at high temperature [37]. On the other hand, for all the oxide-doped compositions, the characteristic temperatures of permittivity peaks agree with that of loss peaks well. Especially, SCBT-Cr shows the most flat dielectric loss curve at 100 Hz, which indicates that the synergetic doping of CeO_2_ and Cr_2_O_3_ could significantly improve the temperature stability of the dielectric properties of SBT.

### 3.3. Electro-Mechanical Coupling Property

**Figure 4:**
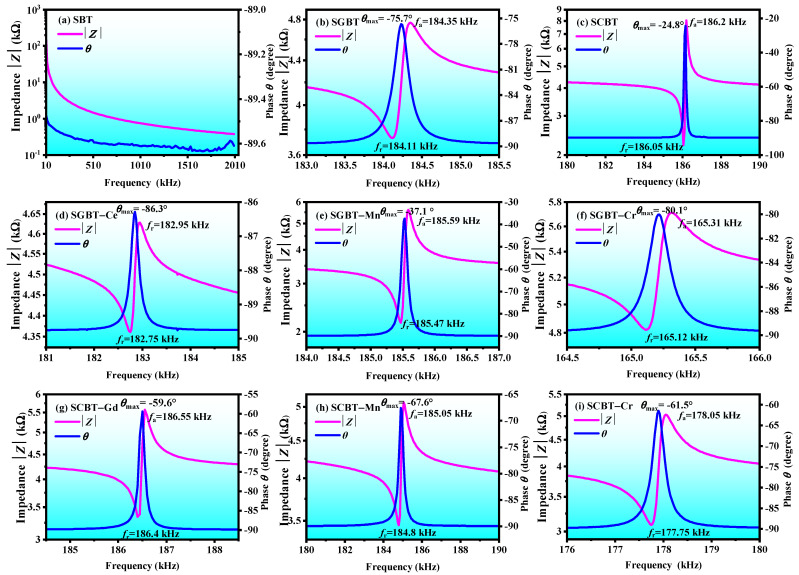
Electro-mechanical resonance spectroscopy of the oxide-modified SBT ceramics at room temperature.

Figure 4 shows the electro-mechanical resonance spectroscopy of the oxide-modified SBT ceramics. As can be seen, there are no resonance-antiresonance peaks in the pure SBT ceramic at the measured frequency range from 20 Hz to 2 MHz. The resonance-antiresonance peaks of SGBT and SCBT appear, respectively, at 184 kHz and 186 kHz. A high angle indicates the fully poled state of the specimen [38]. The position generated the resonance-antiresonance peak and the maximum phase angle also converts with the introduction of other additives. SCBT obtaind the maximum phase angle value (*θ* = −24.8°), which indicates its more fully polarized degree.

Table 3 presents electro-mechanical properties of the oxide-modified SBT ceramics. Clearly, oxide additives also affect the electro-mechanical coupling properties of the SBT ceramic, especially as the addition of Cr_2_O_3_ has a significant impact on it. SGBT-Cr and SCBT-Cr obtain relatively high *k*_p_, low *Q*_m_ and *N*_p_. The oxygen vacancies in piezoceramics usually result in the increase in *Q*_m_ and the decrease in *k*_p_ for ferroelectric ceramics [39]. A higher *k*_p_ achieved by SGBT-Cr and SCBT-Cr can be attributed to the reduced oxygen vacancy concentration caused by the addition of Gd_2_O_3_ and CeO_2_.

### 3.4. Lower Limiting Frequency

**Figure 5:**
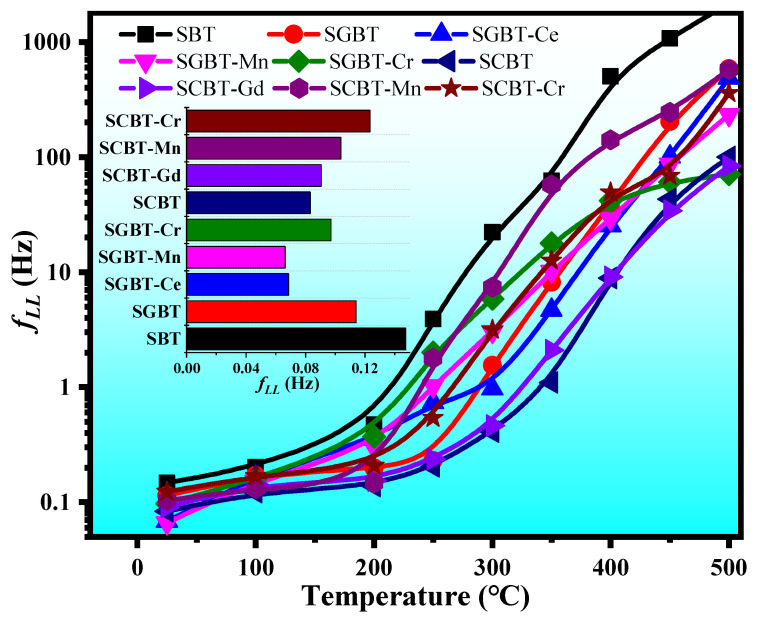
Lower limiting frequency of the oxide-modified SBT ceramics at different temperatures (the insert shows the *f_LL_* values of various compositions at room temperature).

Piezoelectric ceramic materials not only generate charges under the condition of applied stress or strain, but also ensure that the charges must be maintained for a period of time to be monitored by the system in actual engineering applications. The time of the maintained charge is proportional to the *RC* time constant. The minimum available frequency of sensor is considered to be the lower limiting frequency (*f_LL_*). The relationship between *RC* time constant and *f_LL_* is as follows:

(2) $$f_{LL}=\frac{1}{2\pi RC}$$

where *C* is the capacitance (1 kHz) and R is the insulation resistance. Low values of *f_LL_* allow the dynamic bandwidth to be extended to sonic frequencies [40]. The addition of different oxides decreases the *f_LL_* of SBT as shown in the inset of Figure 5 at room temperature. The result indicates that the addition of oxides could improve the resistivity of SBT ceramics. Due to superfluous electrons generated by higher valence, Gd^3+^ and Ce^4+^ substituted lower valence Sr^2+^ can neutralize the oxygen vacancies, which increases the resistivity of SBT. The lower limiting frequency of the oxide-modified SBT ceramics at different temperatures are also compared with each other in Figure 5. The *f_LL_* values of all compositions gradually increase with the rise in temperature, which may be attributed to the decrease in resistivity of the samples with increasing temperature. SCBT shows a lower *f_LL_* value in the measured temperature range as compared to others. High resistivity can prevent applied electrical signals from leaking away in the process of using, only the modified SBT ceramics with high resistivity can be used in high-temperature piezoelectric fields.

### 3.5. Piezoelectric Properties

**Figure 6:**
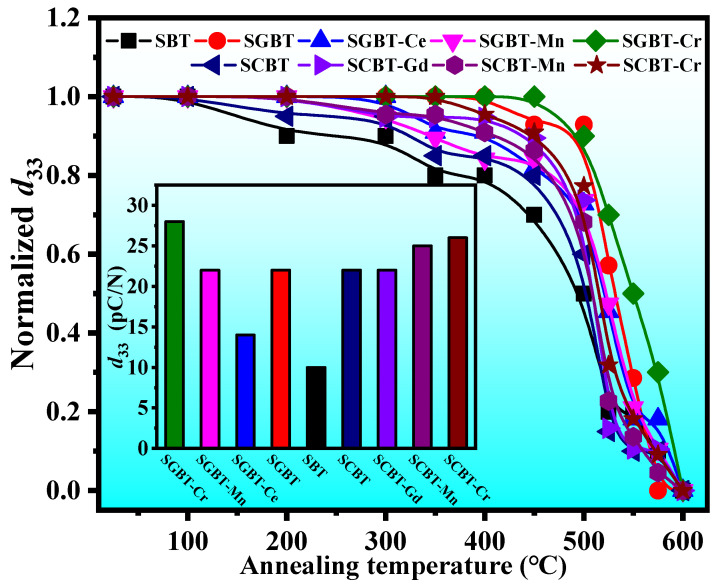
Thermal stability of piezoelectricity of the oxide-modified SBT ceramics (the insert shows the *d***_33_** values of various compositions at room temperature).

The thermal stability of the piezoelectricity of the oxide-modified SBT ceramics is displayed in Figure 6. As can be seen from the insert, before annealing, the piezoelectric properties of the SBT ceramic (*d*_33_ = 10 pC/N) can be improved notably by adding only one of CeO_2_ and Gd_2_O_3_, a higher *d*_33_ ~ 22 pC/N was achieved in SCBT and SGBT. When considering that the addition of CeO_2_ and Gd_2_O_3_ could reduce the concentration of oxygen vacancies as mentioned above, thus the less pinning of domain walls and the elevated resistivity tend to promote the sufficient orientation of ferroelectric domains along the applied electric field during polarization. It is noteworthy that the addition of Cr_2_O_3_ can further enhance the piezoelectric properties of the SBT ceramic that *d*_33_ up to 28 pC/N was observed for SGBT-Cr and 26 pC/N for SCBT-Cr. As shown in Table 1, a larger orthorhombic distortion is obtained for SGBT-Cr and SCBT-Cr, in which a larger spontaneous polarization is believed to form [33]. Further, the thermal stability of piezoelectricity of the oxide-modified SBT ceramics was investigated by the annealing experiment. In general, the *d*_33_ values of all compositions slowly decrease with increasing the annealing temperature from room temperature to 400 °C, and then drastically drop after 400 °C, until they reach zero when the annealing temperature exceeded their *T*_C_. The thermal degradation of piezoelectricity can be attributed to the decoupling of space charges at moderate temperatures and the depolarization of intrinsic dipoles at high temperatures [41]. It should be noted that the *d*_33_ values of SGBT-Cr were decreased by only 3.6% after being annealed at 500 °C and by 18% after being annealed at 550 °C (which is approaching *T*_C_). This result indicates the composition with a good thermally stable piezoelectricity.

In final, dielectric and piezoelectric properties of the oxide-modified SBT ceramics were summarized in Table 4. The high piezoelectric constant, low dielectric loss, and high Curie temperature presented by some compositions demonstrated the successful modification on the SBT ceramic applied by the oxides. As compared to the modified SBT ceramics reported by other works [20,23,25], the optimized composition SGBT-Cr also possesses the competitive electrical properties with a combination of high *T*_C_ ~ 555 °C and a high *d*_33_ ~ 28 pC/N.

## 4. Conclusions

The effects of oxide additives (Gd_2_O_3_, CeO_2_, MnO_2_ and Cr_2_O_3_) on the phase structures and electrical properties of the SBT ceramics were investigated in this work, some main results were obtained as follows: XRD patterns demonstrated that all the oxide-modified SBT ceramics were a single SrBi_4_Ti_4_O_15_ phase. The SBT ceramics with the addition of MnO_2_ presented a high relative density up to 97%. The addition of Gd_2_O_3_ increased the *T*_C_ of SBT, which can be related to the larger orthorhombic distortion caused by the substitution of Gd^3+^ with a smaller ionic radius for Sr^2+^ at A-site. In addition, the addition of CeO_2_ reduced the *T*_C_ of SBT, based on the fact that the stability of oxygen octahedron tends to be weakened by Ce^4+^ with higher coordination number substituting for Sr^2+^ at A-site. The synergetic doping of CeO_2_ and Cr_2_O_3_ could significantly improve the temperature stability of the dielectric properties of SBT. Cr_2_O_3_ can significantly enhance the *k*_p_ of SBT, at the same time, the addition of these oxides also reduced the *f_LL_* of SBT at high temperatures. The addition of oxides could improve the piezoelectric property of SBT (*d*_33_ = 10 pC/N); in particular, SCBT-Cr and SGBT-Cr obtained a higher *d*_33_ of 26 pC/N and 28 pC/N, respectively. Among these compositions, SGBT-Cr (Sr_0.92_Gd_0.053_Bi_4_Ti_4_O_15_ + 0.2 wt% Cr_2_O_3_) presented the best electrical properties, such as: *T*_C_ = 555 °C, *tan δ =* 0.4%, *k*_p_ = 6.35%, *d*_33_ = 28 pC/N, as well as a good thermally stable piezoelectricity that the values of *d*_33_ was decreased by only 3.6% after being annealed at 500 °C for 4 h and retained 82% after being annealed at the temperature approaching *T*_C_.

## Figures and Tables

**Table 1:** Lattice parameters of the oxide-modified SBT ceramics.

Compositions		SBT	SGBT	SGBT-Ce	SGBT-Mn	SGBT-Cr	SCBT	SCBT-Gd	SCBT-Mn	SCBT-Cr
		Orthorhombic, *Bb21m*								
	
*a* (Å)		5.43177	5.42744	5.42855	5.42849	5.42483	5.42807	5.42893	5.42653	5.42411
*b* (Å)		5.43718	5.43507	5.43741	5.43693	5.43767	5.43818	5.43877	5.43772	5.43598
*c* (Å)		40.95524	40.93938	40.92796	40.88468	40.89918	40.95129	40.9501	40.95103	40.93853
*V* (Å^3^)		1209.54	1207.65	1208.08	1206.68	1206.46	1208.83	1209.12	1208.38	1207.85
*b/a*		1.0010	1.0014	1.0016	1.00155	1.0024	1.0019	1.0018	1.0020	1.0022

**Table 2:** Density data of the oxide-modified SBT ceramics.

Compositions	SBT	SGBT	SGBT-Ce	SGBT-Mn	SGBT-Cr	SCBT	SCBT-Gd	SCBT-Mn	SCBT-Cr
*ρ*_theoretic_ (g/cm^3^)	7.4456	7.4667	7.4614	7.4724	7.4522	7.441	7.4292	7.446	7.4367
*ρ*_actual_ (g/cm^3^)	7.0598	6.8822	7.137	7.0692	6.3155	7.1173	7.08528	7.2209	6.5354
*ρ*_relative_ (%)	94.8	92.2	95.7	95.7	84.7	95.6	95.4	97	87.8

**Table 3:** Electro-mechanical properties of the oxide-modified SBT ceramics.

Compositions	SGBT	SGBT-Ce	SGBT-Mn	SGBT-Cr	SCBT	SCBT-Gd	SCBT-Mn	SCBT-Cr
*k*_p_ (%)	5.72	5.2	4.03	6.35	3.7	4.5	5.8	6.51
*Q*_m_	423	465	1240	355	1885	787	443	374
*N*_p_ (Hz·m)	2655	2745	2711	2481	2751	2740	2694	2613

**Table 4:** Comparison of electrical properties between the oxide-modified SBT ceramics and other compositions reported.

Compositions	*ε*_r_ (1 kHz)	*tan δ* (1 kHz)	*T*_c_ (°C)	*d*_33_ (pC/N)
SBT	152	0.9	537	10
SGBT	145	0.4	557	22
SGBT-Ce	168	0.4	552	14
SGBT-Mn	156	0.3	551	22
SGBT-Cr	156	0.3	555	28
SCBT	175	0.1	531	22
SCBT-Gd	169	0.2	539	22
SCBT-Mn	176	0.2	539	24
SCBT-Cr	124	0.3	542	26
SBT-Sm [20]	220	2.0	520	20
SBT-3Co [23]	200	0.6	528	28
SBT-4Mn [25]	180	0.8	530	30

## Data Availability

The data presented in this paper can be provided at the request of the corresponding authors.

## References

[1] Ali F., Raza W., Li X., Gul H., Kim K.H. (2019). Piezoelectric energy harvesters for biomedical applications. Nano Energy.

[2] Song K., Zhao R., Wang Z.L., Yang Y. (2019). Conjuncted pyro-piezoelectric effect for self-powered simultaneous temperature and pressure sensing. Adv. Mater..

[3] Sahu M., Hajra S., Lee K., Deepti P.L., Mistewicz K., Kim H.J. (2021). Piezoelectric nanogenerator based on lead-free flexible PVDF-barium titanate composite films for driving low power electronics. Crystals.

[4] Kim K., Zhang S., Salazar G., Jiang X. (2012). Design, fabrication and characterization of high temperature piezoelectric vibration sensor using YCOB crystals. Sens. Actuators A Phys..

[5] Parks D.A., Zhang S., Tittmann B.R. (2013). High-temperature (>500 °C) ultrasonic transducers: An experimental comparison among three candidate piezoelectric materials. IEEE Trans. Ultrason. Ferroelectr. Freq. Control.

[6] Shrout T.R., Zhang S.J. (2007). Lead-free piezoelectric ceramics: Alternatives for PZT?. J. Electroceram..

[7] Glaum J., Hoffman M. (2014). Electric fatigue of lead-free piezoelectric materials. J. Am. Ceram. Soc..

[8] Vázquez-Rodríguez M., Jiménez F.J., Pardo L., Ochoa P., González A.M., de Frutos J. (2019). A new prospect in road traffic energy harvesting using lead-free piezoceramics. Materials.

[9] De Araujo A.P., Cuchiaro J.D., Mcmillan L.D., Scott M.C., Scott J.F. (1995). Fatigue-free ferroelectric capacitors with platinum electrodes. Nature.

[10] Zhang L., Wang W., Zhou L., Xu H. (2010). Bi_2_WO_6_ nano- and microstructures: Shape control and associated visible-light-driven photocatalytic activities. Small.

[11] Wolfe R.W., Newnham R.E., Smithf D.K., Kay M.I. (1972). Crystal structure of Bi_3_TiNbO_9_. Ferroelectrics.

[12] Chen Y., Liang D., Wang Q., Zhu J. (2014). Microstructures, dielectric, and piezoelectric properties of W/Cr co-doped Bi_4_Ti_3_O_12_ ceramics. J. Appl. Phys..

[13] Zhang S.T., Sun B., Yang B. (2001). SrBi_4_Ti_4_O_15_ thin films of Ti containing bismuth-layered-ferroelectrics prepared by pulsed laser deposition. Mater. Lett..

[14] Ferrer P., Algueró M., Iglesias J.E., Castro A. (2007). Processing and dielectric properties of Bi_4_Sr_n−3_Ti_n_O_3n+3_ (n = 3, 4 and 5) ceramics obtained from mechanochemically activated precursors. J. Eur. Ceram. Soc..

[15] Stevenson T., Martin D.G., Cowin P.I., Blumfield A., Bell A.J., Comyn T.P., Weaver P.M. (2015). Piezoelectric materials for high temperature transducers and actuators. J. Mater. Sci. Mater. Electron..

[16] Nalini G., Row T. (2002). Structure determination at room temperature and phase transition studies above *T*_c_ in ABi_4_Ti_4_O_15_ (A = Ba, Sr or Pb). Bull. Mater. Sci..

[17] Nayak P., Badapanda T., Panigrahi S. (2016). Dielectric, ferroelectric and conduction behavior of tungsten modified SrBi_4_Ti_4_O_15_ ceramic. J. Mater. Sci. Mater. Electron..

[18] Ramana E.V., Graca M.P.F., Valente M.A., Sankaram T.B. (2014). Improved ferroelectric and pyroelectric properties of Pb-doped SrBi_4_Ti_4_O_15_ ceramics for high temperature applications. J. Alloys Compd..

[19] Rajashekhar G., Sreekanth T., James A.R., Ravi Kiran U., Sarah P. (2020). Dielectric properties of sodium and neodymium substitute to A-Site SrBi_4_Ti_4_O_15_ ceramics. Ferroelectrics.

[20] Yu L., Hao J., Xu Z., Li W., Chu R. (2017). Reddish orange-emitting and improved electrical properties of Sm_2_O_3_-doped SrBi_4_Ti_4_O_15_ multifunctional ceramics. J. Mater. Sci. Mater. Electron..

[21] Nayak P., Badapanda T., Panigrahi S. (2016). Dielectric and ferroelectric properties of Lanthanum modified SrBi_4_Ti_4_O_15_ ceramics. Mater. Lett..

[22] Nayak P., Badapanda T., Singh A.K., Panigrahi S. (2017). Possible relaxation and conduction mechanism in W^6+^ doped SrBi_4_Ti_4_O_15_ ceramic. Ceram. Int..

[23] Wang Q., Cao Z.P., Wang C.M., Fu Q.W., Yin D.F., Tian H.H. (2016). Thermal stabilities of electromechanical properties in cobalt-modified strontium bismuth titanate (SrBi_4_Ti_4_O_15_). J. Alloys Compd..

[24] Hua H., Liu H., Ouyang S. (2009). Structure and ferroelectric property of Nb-doped SrBi_4_Ti_4_O_15_ ceramics. J. Electroceram..

[25] Cao Z.P., Wang C.M., Lau K., Wang Q., Fu Q.W., Tian H.H., Yin D.F. (2016). Large enhancement of piezoelectric properties in Mn-modified SrBi_4_Ti_4_O_15_ and its thermal stabilities at elevated temperatures. Ceram. Int..

[26] Zhao T.L., Wang C.M., Wang C.L., Wang Y.M., Dong S. (2015). Enhanced piezoelectric properties and excellent thermal stabilities of cobalt-modified Aurivillius-type calcium bismuth titanate (CaBi_4_Ti_4_O_15_). Mater. Sci. Eng. B.

[27] Rajashekhar G., Sreekanth T., Ravikiran U., Sarah P. (2020). Dielectric properties of Na and Pr doped SrBi_4_Ti_4_O_15_ ceramics. Mater. Today Proc..

[28] Du H., Ma C., Ma W., Wang H. (2018). Microstructure evolution and dielectric properties of Ce-doped SrBi_4_Ti_4_O_15_ ceramics synthesized via glycine-nitrate process. Process. Appl. Ceram..

[29] Liang C.K., Long W. (2010). Microstructure and properties of Cr_2_O_3_-doped ternary lead zirconate titanate ceramics. J. Am. Ceram. Soc..

[30] Wang C.M., Wang J.F. (2006). High performance Aurivillius phase sodium-potassium bismuth titanate lead-free piezoelectric ceramics with lithium and cerium modification. Appl. Phys. Lett..

[31] Chen Y., Xie S., Wang Q., Zhu J. (2016). Influence of Cr_2_O_3_ additive and sintering temperature on the structural characteristics and piezoelectric properties of Bi_4_Ti_2.95_W_0.05_O_12.05_ Aurivillius ceramics. Prog. Nat. Sci. Mater. Int..

[32] Newnham R.E. (1967). Cation ordering in Na_0.5_Bi_4.5_Ti_4_O_15_. Mater. Res. Bull..

[33] Chen Y., Pen Z., Wang Q., Zhu J. (2014). Crystalline structure, ferroelectric properties, and electrical conduction characteristics of W/Cr co-doped Bi_4_Ti_3_O_12_ ceramics. J. Alloys Compd..

[34] Chen Y., Xu J., Xie S., Tan Z., Nie R., Guan Z., Wang Q., Zhu J. (2018). Ion Doping effects on the lattice distortion and interlayer mismatch of aurivillius-type bismuth titanate compounds. Materials.

[35] Kumar S., Varma K. (2010). Structural, dielectric and ferroelectric properties of four-layer Aurivillius phase Na_0.5_La_0.5_Bi_4_Ti_4_O_15_. Mater. Sci. Eng. B.

[36] Shulman H.S., Damjanovic D., Setter N. (2010). Niobium doping and dielectric anomalies in bismuth titanate. J. Am. Ceram. Soc..

[37] Chen Y., Xie S., Wang H., Chen Q., Wang Q., Zhu J., Guan Z. (2017). Dielectric abnormality and ferroelectric asymmetry in W/Cr co-doped Bi_4_Ti_3_O_12_ ceramics based on the effect of defect dipoles. J. Alloys Compd..

[38] Chen Y., Wang S., Zhou H., Xu Q., Wang Q., Zhu J. (2020). A systematic analysis of the radial resonance frequency spectra of the PZT-based (Zr/Ti = 52/48) piezoceramic thin disks. J. Adv. Ceram..

[39] Hou Y.D., Lu P.X., Zhu M.K., Song X.M., Tang J.L., Wang B., Yan H. (2005). Effect of Cr_2_O_3_ addition on the structure and electrical properties of Pb((Zn_1/3_Nb_2/3_)_0.20_(Zr_0.50_Ti_0.50_)_0.80_)O_3_ ceramics. Mater. Sci. Eng. B.

[40] Turner R.C., Fuierer P.A., Newnham R.E., Shrout T.R. (1994). Materials for high-temperature acoustic and vibration sensors—A review. Appl. Acoust..

[41] Chun P. (1997). Influence of mobile space charges on the ferroelectric properties of (K_0.50_Na_0.50_)_2_(Sr_0.75_Ba_0.25_)_4_Nb_10_O_30_ ceramics. J. Appl. Phys..

